# Functional Roles of NOD1 in Odontoblasts on Dental Pulp Innate Immunity

**DOI:** 10.1155/2016/9325436

**Published:** 2016-09-25

**Authors:** Yuki Hosokawa, Kouji Hirao, Hiromichi Yumoto, Ayako Washio, Tadashi Nakanishi, Daisuke Takegawa, Chiaki Kitamura, Takashi Matsuo

**Affiliations:** ^1^Department of Conservative Dentistry, Institute of Biomedical Sciences, Tokushima University Graduate School, 3-18-15 Kuramoto-cho, Tokushima 770-8504, Japan; ^2^Division of Endodontics and Restorative Dentistry, Department of Oral Functions, Kyushu Dental University, 2-6-1 Manazuru, Kokura-kita, Kitakyushu, Fukuoka 803-8580, Japan

## Abstract

Caries-related pathogens are first recognized by odontoblasts and induce inflammatory events that develop to pulpitis. Generally, initial sensing of microbial pathogens is mediated by pattern recognition receptors, such as Toll-like receptor and nucleotide-binding oligomerization domain (NOD); however, little is known about NODs in odontoblasts. In this study, the levels of NODs expressed in rat odontoblastic cell line, KN-3, were assessed by flow cytometry and the levels of chemokines in NOD-specific ligand-stimulated KN-3 cells were analyzed by real-time PCR and ELISA. The signal transduction pathway activated with NOD-specific ligand was assessed by blocking assay with specific inhibitors and reporter assay. In KN-3 cells, the expression level of NOD1 was stronger than that of NOD2 and the production of chemokines, such as CINC-1, CINC-2, CCL20, and MCP-1, was upregulated by stimulation with NOD1-specific ligand, but not with NOD2-specific ligand. CINC-2 and CCL20 production by stimulation with NOD1-specific ligand was reduced by p38 MAPK and AP-1 signaling inhibitors. Furthermore, the reporter assay demonstrated AP-1 activation in NOD1-specific ligand-stimulated KN-3 cells. These findings indicated that NOD1 expressed in odontoblasts functions to upregulate the chemokines expression via p38-AP-1 signaling pathway and suggested that NOD1 may play important roles in the initiation and progression of pulpitis.

## 1. Introduction

Pulpitis, inflammation of the dental pulp, is mainly caused by the dental caries-related pathogens invading into dentinal tubules as well as mechanical and chemical irritations. Regarding the initiation of this inflammatory disease, odontoblasts located in the outermost layer in dental pulp first recognize caries-related pathogens, sense such external irritations, and play important roles in the innate immune system of dental pulp tissues. Generally, the initial sensing of microbial pathogens is mediated by pattern recognition receptors (PRRs), such as Toll-like receptor (TLR) and the nucleotide-binding oligomerization domain (NOD), for pathogen-associated molecular patterns (PAMPs). TLR2, which is a cell surface receptor, is crucial for the recognition of peptidoglycan, lipoprotein, and lipoteichoic acid (LTA), whereas cell surface receptor TLR4 plays a major role in the detection of lipopolysaccharide (LPS) [1]. Besides TLRs, NODs are also innate immunity receptors, but they are localized intracellularly. NOD1 and NOD2 recognize active entities of peptidoglycan containing *γ*-D-diaminopimelic acid (iE-DAP) and muramyldipeptide (MDP), respectively [2, 3].

In inflamed dental pulpal tissues, the expression levels of various chemokines, such as CCL2, CCL3, CCL23, and interleukin- (IL-) 8 (CXCL8), were detected in the odontoblastic layer [4–6]. Similarly, cultured odontoblastic cells express chemokines, including CCL2, CCL26, CXCL4, CXCL8, CXCL12, and CXCL14, and chemokine receptors, such as CXCR2, CCRL1, and CCRL2 [5, 7]. We previously demonstrated that TLR2 and NOD2 were the functionally predominant receptors stimulating the production of proinflammatory mediators, such as IL-8, IL-6, and monocyte-chemoattractant protein- (MCP-) 1 on cultured human dental pulp fibroblasts (HDPFs), and that mitogen-activated protein kinase (MAPK) and nuclear factor-*κ*B (NF-*κ*B) were involved in stimulated HDPF signals [8, 9]. Previous studies have reported that odontoblast-like cells differentiated* in vitro* from human dental pulp explants express TLR1-6 and TLR9 genes [5], and odontoblasts* in situ* express TLR2 and TLR4 on their cellular processes and cell surfaces [10]. Moreover, recent studies showed that NOD1 expression was weakly positive in the cytoplasm of odontoblasts in specimens without carious lesion but was upregulated in the odontoblastic layer of specimens with carious lesion [11], and NOD2 protein was distinctly expressed in the cytoplasm of odontoblasts [12]. However, little is known about the expression levels and functions of NODs in odontoblasts. Hence, we focused on the functional roles of NODs and the cell signaling pathways through NODs in odontoblasts. Primary odontoblasts have difficulty in passage culture because of replicative senescence and the incomplete odontoblastic properties of dental pulp cell lines [13]. To solve this problem, KN-3 cells, a rat odontoblastic cell line, have been established, and a previous report demonstrated that KN-3 cells have high levels of alkaline phosphatase activity, express odontoblastic cell markers [14–16], such as dentine sialophosphoprotein and Runt-related transcription factor (Runx)2 [13], dentin matrix protein-1 [17], and form mineralized nodules [13]. Furthermore, a recent report showed that KN-3 cells are a representative authentic control of odontoblast-like cells derived from iPS cells [18, 19] and indicated the importance of KN-3 to investigate odontoblasts and dental pulpal inflammation. Therefore, we used KN-3 cells as representative of authentic odontoblasts in this study. In this study, we first confirmed the expression levels of NODs in rat KN-3 cells to determine the effects of NODs on the induction of proinflammatory chemokines and further investigate the cell signaling pathways in PAMP-stimulated KN-3 cells.

## 2. Materials and Methods

### 2.1. Cell Culture

A rat clonal odontoblast-like cell line, KN-3, was cultured in minimum essential medium alpha (Life Technologies, Carlsbad, CA, USA) containing 10% fetal bovine serum (Sigma-Aldrich, St. Louis, MO, USA), 100 U mL^−1^ penicillin, and 100 *μ*g mL^−1^ streptomycin (Life Technologies) at 37°C in a humidified atmosphere of 5% CO_2_. Approximately 8 × 10^4^ cells mL^−1^ in medium were seeded in wells of 24- or 6-well tissue culture plates and incubated until confluent monolayers developed. Confluent KN-3 monolayers were used in all experiments.

### 2.2. Reagents

iE-DAP and *γ*-D-glutamyl-Lysine (iE-Lys) were purchased from InvivoGen (San Diego, CA, USA). MDP was purchased from Sigma-Aldrich. An inactive stereoisomer of MDP,* N*-acetylmuramyl-L-alanyl-L-isoglutamine (MDP-LL), was purchased from BACHEM (Bubendorf, Switzerland). Recombinant rat tumor necrosis factor- (TNF-) *α* was obtained from PEPROTECH (Rocky Hill, NJ, USA). PD98059 and SP600125 were purchased from Merck Biosciences Ltd. (Darmstadt, Germany). SB203580 and SN50 were obtained from Santa Cruz Biotechnology (Santa Cruz, CA, USA) and Enzo Life Sciences (Farmingdale, NY, USA), respectively. SR11302 was purchased from Tocris Bioscience (Bristol, UK).

### 2.3. Flow Cytometry

KN-3 cells fixed in 4% paraformaldehyde were treated with BD Cytofix/Cytoperm solution (BD Biosciences, San Jose, CA, USA), incubated with anti-rat NOD1 (NOVUS BIOLOGICALS, Littleton, CO, USA), NOD2 (Abnova, Jhouzih St., Taipei, Taiwan) antibodies or their respective isotype-matched controls followed by reaction with fluorescein isothiocyanate- (FITC-) conjugated rabbit IgG secondary antibody (Dako, Carpinteria, CA, USA). Stained cells were analyzed by flow cytometry (EPICS XL; Beckman Coulter, Hialeah, FL, USA).

### 2.4. Cytokine Antibody Array and Enzyme-Linked Immunosorbent Assay

KN-3 monolayers in 24-well tissue culture plates were stimulated with iE-DAP (1 or 10 *μ*g mL^−1^), iE-Lys (10 *μ*g mL^−1^), MDP (1 or 10 *μ*g mL^−1^), MDP-LL (10 *μ*g mL^−1^), or TNF-*α* (0.01 *μ*g mL^−1^) for 24 h. The concentrations of these NOD ligands were decided by reference to the manufacturer's instructions as well as our previous study for HDPFs [8]. The detection of cytokines and chemokines in cell culture supernatants obtained from stimulated and unstimulated KN-3 cells was performed using Rat Cytokine Array C2 (RayBiotech, Inc., Norcross, GA, USA) in accordance with the manufacturer's instructions. The concentrations of chemokines in cell culture supernatants were quantified using commercially available enzyme-linked immunosorbent assay (ELISA) kits (for CCL20, CINC-1, and CINC-2; R&D Systems, Minneapolis, MN, USA: for MCP-1; PEPROTECH) in accordance with the manufacturer's instructions.

### 2.5. Analysis of PRR-Specific Ligand- or TNF-*α*-Stimulated Cell Signal Transduction Pathways

KN-3 monolayers in 24-well tissue culture plates were pretreated with specific inhibitors: PD98059 (for extracellular signal-regulated kinase [ERK] 1/2; 25 *μ*M), SB203580 (for p38 MAPK; 10 *μ*M), SP600125 (for c-jun NH_2_-terminal kinase [JNK]; 10 *μ*M), SN-50 (for NF-*κ*B inhibitor; 9 *μ*M), or SR11302 (activator protein [AP]-1 signaling inhibitor; 10 *μ*M) for 1 h prior to stimulation. The concentrations of these specific inhibitors were decided by reference to the data sheets from the vendors for inhibitors as well as our previous results [9]. We also confirmed that all inhibitors at the indicated concentration have no cytotoxic effect on the viability of KN-3 cells by LDH cytotoxicity assay (data not shown).

### 2.6. Real-Time Reverse Transcription-Polymerase Chain Reaction

KN-3 monolayers in 24-well tissue culture plates were stimulated with iE-DAP (1 or 10 *μ*g mL^−1^), iE-Lys (10 *μ*g mL^−1^), MDP (1 or 10 *μ*g mL^−1^), MDP-LL (10 *μ*g mL^−1^), or TNF-*α* (0.01 *μ*g mL^−1^) for the indicated periods. Total RNA from KN-3 cells was isolated using a NucleoSpin RNA kit (MACHEREY-NAGEL, Duren, Germany), and 20 ng RNA was utilized for each real-time reverse transcription-polymerase chain reaction (RT-PCR). RT and real-time PCR were performed in two steps, as follows. cDNA synthesis was performed using PrimeScript RT Master Mix (TaKaRa, Shiga, Japan), and specific gene transcriptions were amplified using Fast SYBR Green Master Mix and a StepOnePlus Real-Time PCR System (Thermo Fisher Scientific, Waltham, MA, USA). A housekeeping gene, glyceraldehyde-3-phosphate dehydrogenase (GAPDH), was used for sample normalization. The designs of PCR primers are shown in Table 1. For each target gene, relative expression was determined after normalization using the ΔΔCt method. Results were expressed as fold-change values relative to unstimulated control samples.

### 2.7. Sodium Dodecyl Sulfate-Polyacrylamide Gel Electrophoresis and Immunoblotting Analysis

KN-3 monolayers in 6-well tissue culture plates were stimulated with iE-DAP (10 *μ*g mL^−1^), iE-Lys (10 *μ*g mL^−1^), or TNF-*α* (0.01 *μ*g mL^−1^) for 5 or 10 min. Stimulated or unstimulated KN-3 cells were collected in RIPA lysis buffer (Santa Cruz Biotechnology). The protein concentrations in lysates were quantified using a bicinchoninic acid protein assay kit (Sigma-Aldrich). Equal amount of protein was loaded onto a 5–15% sodium dodecyl sulfate-polyacrylamide gel electrophoresis (SDS-PAGE) gel (Bio-Rad Laboratories, Hercules, CA, USA), followed by electrotransfer to a polyvinylidene difluoride membrane. The membrane was first incubated with inhibitor *κ*B (I*κ*B) *α* antibody (Sigma-Aldrich) or phospho-I*κ*B*α* antibody (Cell Signaling Technology, Danvers, MA, USA). After washing, the membrane was reacted with horseradish peroxidase-conjugated secondary antibody (Sigma-Aldrich). Protein bands were finally visualized on X-ray film with the use of ECL Prime Western Blotting Detection System (GE Healthcare, Buckinghamshire, UK). Actin levels were also assessed using an anti-actin antibody as an internal control (Sigma-Aldrich).

### 2.8. Cell Signal Reporter Assay

A green fluorescent protein (GFP) GFP reporter construct with transcriptional response element for AP-1 or NF-*κ*B was transiently transfected into KN-3 cells, incubated for 24 h, and then subjected to stimulation with iE-DAP (10 *μ*g mL^−1^), iE-Lys (10 *μ*g mL^−1^), or TNF-*α* (0.01 *μ*g mL^−1^) for 24 h. Cell signal transduction pathways in stimulated or unstimulated KN-3 cells were determined using Cignal Reporter Assays (Qiagen, Valencia, CA, USA) in accordance with the manufacturer's instructions.

### 2.9. Statistical Analysis

All statistical analyses were performed using the multifactorial one-way analysis of variance (ANOVA) with Bonferroni post hoc tests to assess differences between multiple sets of data. Differences were considered significant when the probability value was less than 5% (*p* < 0.05).

## 3. Results

### 3.1. Pattern Recognition Receptor Expression in KN-3 Cells

**Figure 1:**
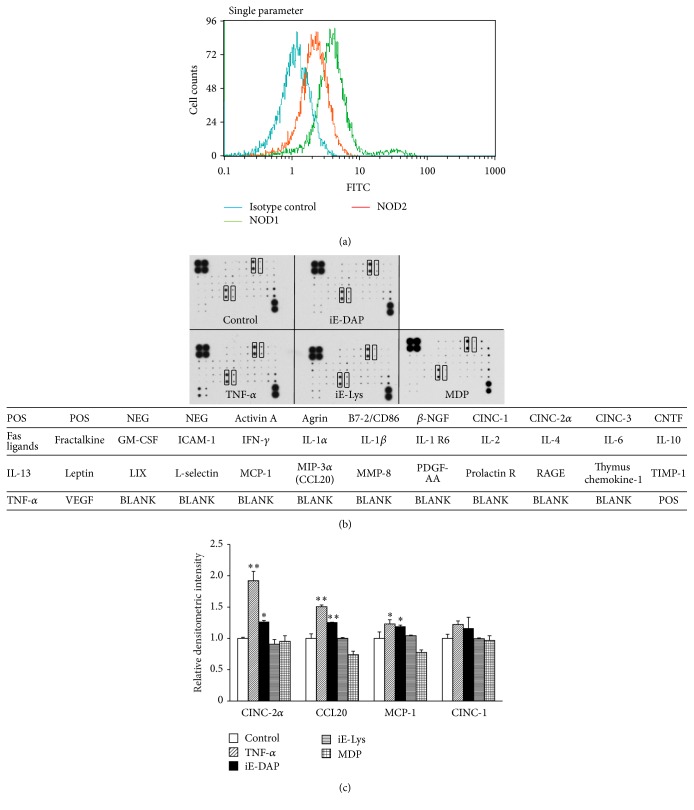
Constitutive expression of NOD1 and NOD2 in rat KN-3 cells and chemokine production in iE-DAP- and TNF-*α*-stimulated KN-3 cells. (a) The constitutive expression of NOD1 and NOD2 in KN-3 cells was assessed by flow cytometry. The results are representative of four different experiments demonstrating similar results. (b, c) KN-3 cells were stimulated with iE-DAP (10 *μ*g mL^−1^), iE-Lys (10 *μ*g mL^−1^), MDP (10 *μ*g mL^−1^), or TNF-*α* (0.01 *μ*g mL^−1^) for 24 h. The detection of cytokines and chemokines produced in the cell culture supernatants was performed using a Rat Cytokine Antibody Array. (b) The results shown are representative images of two independent experiments with similar results. (c) Densitometric analysis of various chemokine production levels. Bars indicate the relative densitometric intensities after the values were normalized with both positive and negative controls and background using ImageJ software. In particular, positive controls were used to normalize the values from different membranes being compared. Values represent the means ± SDs of two independent experiments. Asterisks indicate significant differences (^*∗*^ *p* < 0.05 and ^*∗∗*^ *p* < 0.01) versus nonstimulated control group.

We first investigated whether KN-3 cells express NOD1 and NOD2. Flow cytometric analysis showed that intracellular expression level of NOD1 was stronger than that of NOD2 (Figure 1(a)).

### 3.2. Chemokine Induction in KN-3 Cells Stimulated with NOD-Specific Ligand or TNF-*α*

**Figure 2:**
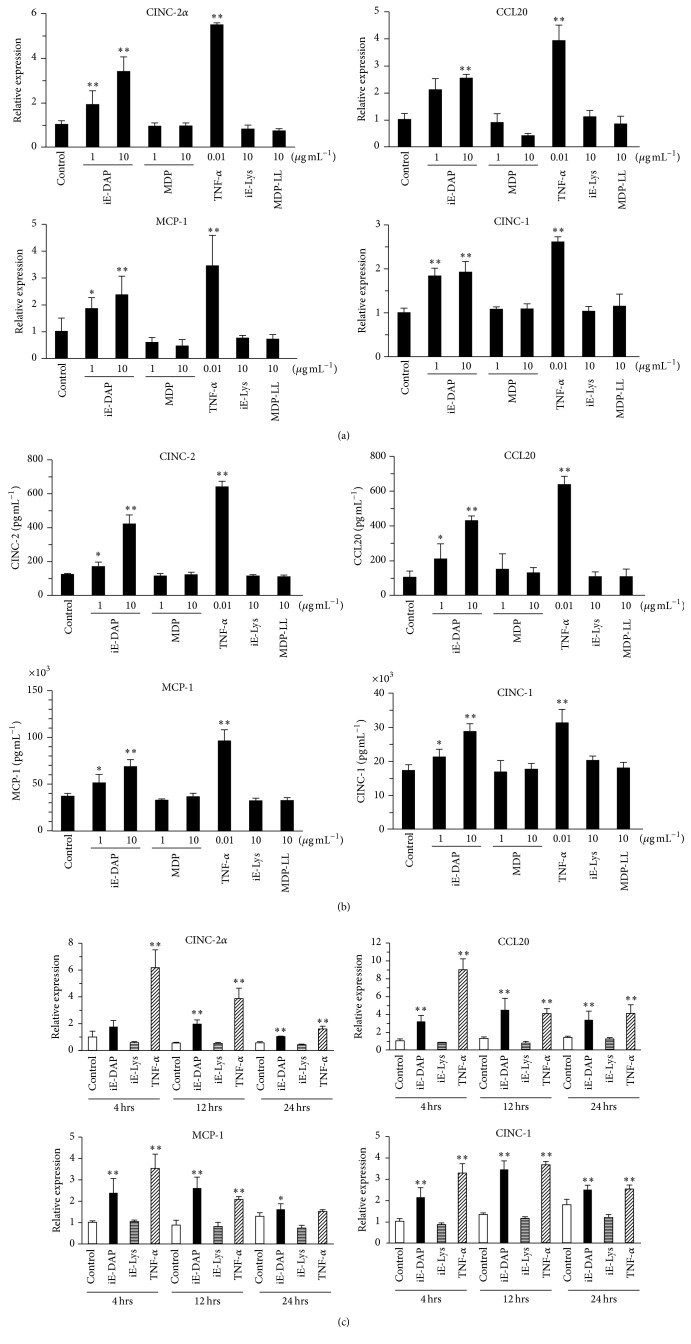
Expression and production of chemokines induced in KN-3 cells stimulated with NOD1 or NOD2 ligand. (a, b) KN-3 cells were stimulated with iE-DAP (1 or 10 *μ*g mL^−1^), iE-Lys (10 *μ*g mL^−1^), MDP (1 or 10 *μ*g mL^−1^), MDP-LL (10 *μ*g mL^−1^), or TNF-*α* (0.01 *μ*g mL^−1^) for 24 h. (a) After stimulation, total RNA was isolated and mRNA expression levels of CINC-1, CINC-2*α*, MCP-1, and CCL20 were analyzed by real-time reverse transcription-polymerase chain reaction (RT-PCR). (b) The concentrations of CINC-1, CINC-2, MCP-1, and CCL20 in the cell culture supernatants were determined by enzyme-linked immunosorbent assay (ELISA). (c) KN-3 cells were stimulated with iE-DAP (10 *μ*g mL^−1^), iE-Lys (10 *μ*g mL^−1^), or TNF-*α* (0.01 *μ*g mL^−1^) for 4, 12, or 24 h. After stimulation, total RNA was isolated and mRNA expression levels of CINC-1, CINC-2*α*, MCP-1, and CCL20 were analyzed by real-time RT-PCR. Values represent the means ± SDs from representative of four independent experiments and each experiment was performed in quadruplicate. Asterisks indicate significant differences (^*∗*^ *p* < 0.05 and ^*∗∗*^ *p* < 0.01) versus nonstimulated control group.

We examined whether NODs constitutively expressed in KN-3 actually function as a receptor to produce cytokines and chemokines by stimulation with NOD-specific ligand. A cytokine antibody array, which is useful to identify the production profiles of multiple cytokines and chemokines, showed that iE-DAP (NOD1 ligand) and TNF-*α* (as a positive control, proinflammatory cytokine) significantly induced the production of CINC-2*α*, CCL20 (macrophage inflammatory protein-3*α*; MIP-3*α*), and MCP-1 in KN-3 cells and indicated an increasing trend in the levels of CINC-1 in iE-DAP- or TNF-*α*-stimulated KN-3 cells (Figures 1(b) and 1(c)); however, cytokine and chemokine levels were not significantly induced by the stimulation of iE-Lys (NOD1 ligand negative control) or MDP (NOD2 ligand). However, the intensity of CINC-1 and MCP-1, which are constitutively produced at a high level, is largely saturated and this array is a semiquantitative measurement, not quantitative method. Next, to quantitate mRNA expression and production levels of these chemokines in KN-3 cells stimulated with each NOD-specific ligand, real-time RT-PCR and ELISA were performed, respectively. iE-DAP and TNF-*α* significantly induced the mRNA expression levels and production of CINC-1, CINC-2*α*, MCP-1, and CCL20. iE-Lys and MDP failed to induce the expression and production of these chemokines (Figures 2(a) and 2(b)). Moreover, the mRNA expression levels of these chemokines increased within 4 h after stimulation with iE-DAP, reached a maximum at 12 h after stimulation, and were still elevated after 24 h of stimulation (Figure 2(c)).

### 3.3. Cell Signaling Pathways in iE-DAP- or TNF-*α*-Stimulated KN-3 Cells

**Figure 3:**
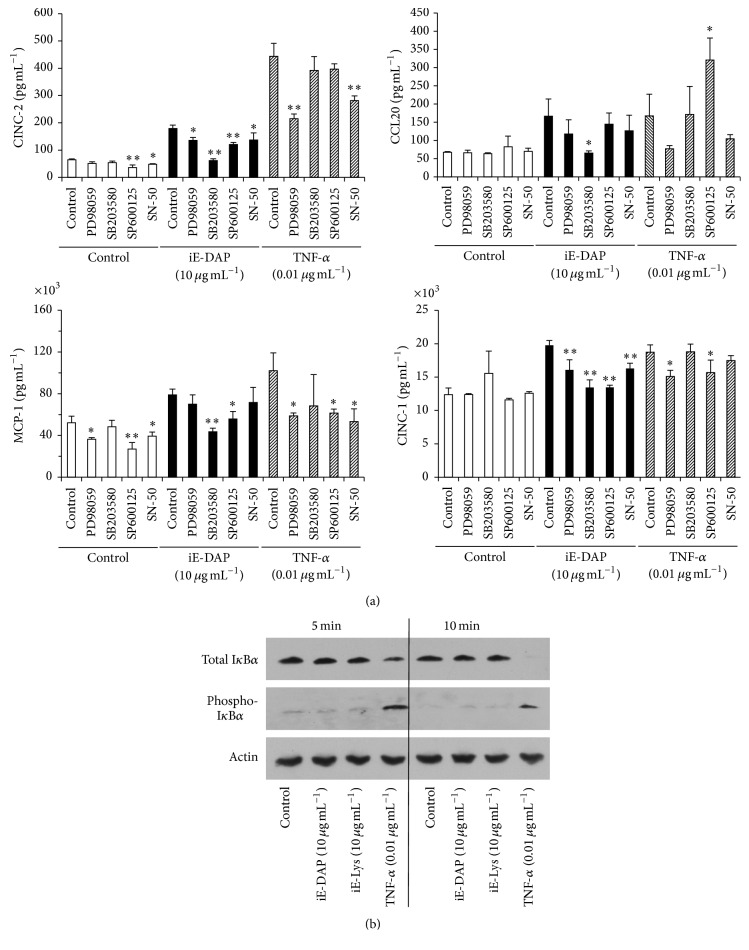
Cell signaling pathway analysis in iE-DAP- and TNF-*α*-stimulated KN-3 cells. (a) KN-3 cells were treated with PD98059 (25 *μ*M), SB203580 (10 *μ*M), SP600125 (10 *μ*M), or SN-50 (9 *μ*M) for 1 h followed by stimulation with iE-DAP (10 *μ*g mL^−1^) or TNF-*α* (0.01 *μ*g mL^−1^) for 24 h. The concentrations of CINC-1, CINC-2, MCP-1, and CCL20 in cell culture supernatants were determined by ELISA. Values represent the means ± SDs from representative of three independent experiments and each experiment was performed in triplicate. Asterisks indicate significant differences (^*∗*^ *p* < 0.05 and ^*∗∗*^ *p* < 0.01) versus nonstimulated control group. (b) Immunoblotting analysis of I*κ*B*α* in nonstimulated control and iE-DAP- or TNF-*α*-stimulated KN-3 cells. KN-3 cells were stimulated with iE-DAP (10 *μ*g mL^−1^), iE-Lys (10 *μ*g mL^−1^), or TNF-*α* (0.01 *μ*g mL^−1^) for 5 or 10 min. Equal loading of gels was confirmed with both sodium dodecyl sulfate-polyacrylamide gel electrophoresis (SDS-PAGE) and immunoblotting using an anti-actin antibody. The results shown are representative images of three independent experiments with similar results.

Cell signaling pathways in iE-DAP- or TNF-*α*-stimulated KN-3 cells were investigated using several specific cell signaling pathway inhibitors. In iE-DAP-stimulated KN-3 cells, CINC-1, CINC-2, CCL20, and MCP-1 production levels were mainly reduced by p38 MAPK inhibitor, SB203580, and JNK inhibitor, SP600125, could slightly inhibit the production of CINC-2, MCP-1, and CINC-1; however NF-*κ*B inhibitor, SN50, could not reduce the production of these chemokines in iE-DAP-stimulated cells except for CINC-2 and CINC-1. On the other hand, in TNF-*α*-stimulated KN-3 cells, ERK 1/2 inhibitor, PD98059, and NF-*κ*B inhibitor, SN50, significantly inhibited the induction of CINC-2 and MCP-1 (Figure 3(a)).

To determine the activation of NF-*κ*B in iE-DAP- or TNF-*α*-stimulated KN-3 cells, the phosphorylation of I*κ*B*α* was analyzed by immunoblotting analysis. The phosphorylation of I*κ*B*α* and the degradation of total I*κ*B*α* were observed in TNF-*α*-stimulated KN-3 cells but not in iE-DAP-stimulated cells (Figure 3(b)).

**Figure 4:**
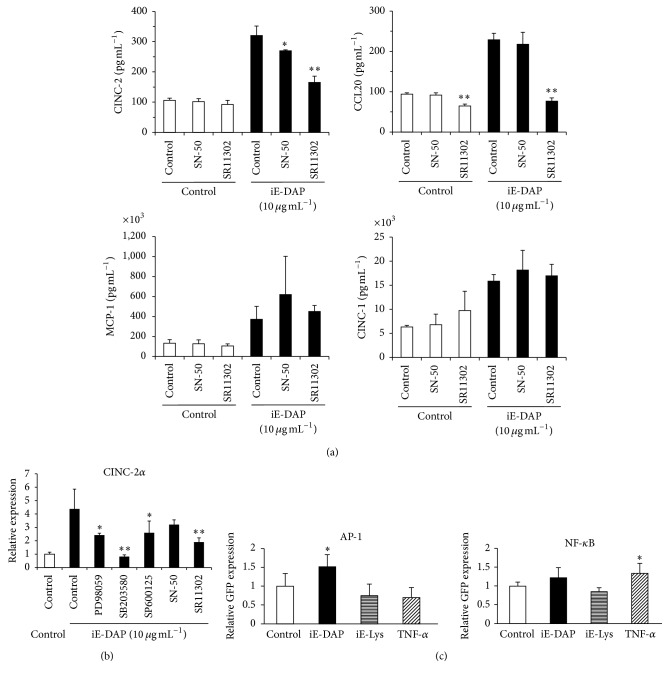
The effects of AP-1 signal pathway in iE-DAP-stimulated KN-3 cells. (a, b) KN-3 cells were treated with PD98059 (25 *μ*M), SB203580 (10 *μ*M), SP600125 (10 *μ*M), SN-50 (9 *μ*M), or SR11302 (10 *μ*M) for 1 h followed by stimulation with iE-DAP (10 *μ*g mL^−1^) for 24 h. (a) The concentrations of CINC-1, CINC-2, MCP-1, and CCL20 in cell culture supernatants were determined by enzyme-linked immunosorbent assay (ELISA). Values represent the means ± SDs from representative of three independent experiments and each experiment was performed in triplicate. Asterisks indicate significant differences (^*∗*^ *p* < 0.05 and ^*∗∗*^ *p* < 0.01) versus noninhibitor control group. (b) mRNA expression level of CINC-2*α* was analyzed by real-time RT-PCR. Values represent the means ± SDs from representative of three independent experiments and each experiment was performed in quadruplicate. Asterisks indicate significant differences (^*∗*^ *p* < 0.05 and ^*∗∗*^ *p* < 0.01) versus noninhibitor control group. (c) A GFP reporter construct with transcriptional response element for AP-1 or NF-*κ*B was transiently transfected into KN-3 cells and subjected to stimulation with iE-DAP (10 *μ*g mL^−1^), iE-Lys (10 *μ*g mL^−1^), or TNF-*α* (0.01 *μ*g mL^−1^) for 24 h. The expression level of GFP was measured using a fluorescence microplate reader (Infinite® 200 PRO, Tecan, Männedorf, Switzerland). Values represent the means ± SDs from representative of three independent experiments and each experiment was performed in quadruplicate. Asterisks indicate significant differences (^*∗*^ *p* < 0.05) versus nonstimulated control group.

It has been reported that NOD1 is essential, not only for NF-*κ*B activation, but also for the activation of MAPKs and AP-1 during* Helicobacter pylori* infection [20]. Therefore, we next examined whether an AP-1 signaling inhibitor, SR11302, can inhibit chemokine production in iE-DAP-stimulated KN-3 cells. In addition to SB203580 (Figure 3(a)), SR11302 significantly inhibited the production of CINC-2 and CCL20, but the production of MCP-1 and CINC-1 was not inhibited by SR11302 in iE-DAP-stimulated KN-3 cells (Figure 4(a)). The mRNA expression level of CINC-2*α* was also significantly reduced by SR11302 and SB203580 and these reduced levels were stronger than those of PD98059 and SP600125 (Figure 4(b)).

To finally determine whether AP-1 or NF-*κ*B, as a downstream transcription factor, is activated in KN-3 cells stimulated with iE-DAP or TNF-*α*, a GFP reporter construct with each transcriptional response element was transiently transfected in KN-3 cells and subjected to stimulation with iE-DAP or TNF-*α*. This reporter assay experiment demonstrated that AP-1 and NF-*κ*B were significantly activated by stimulation with iE-DAP and TNF-*α*, respectively (Figure 4(c)).

## 4. Discussion

Previous reports have shown that human odontoblasts expressed TLR2, TLR3, TLR4, TLR7, TLR8, and TLR9 [21, 22] and TLR2 and TLR4 functioned in human odontoblastic cells [22, 23]. Regarding NODs, previous reports have demonstrated that the expression of NOD2 mRNA was increased in Pam3CSK4 (TLR2-specific ligand)-stimulated human odontoblast-like cells [24], and NOD2 protein was detected by immunohistochemistry in the cytoplasm of odontoblasts [12]. In addition, recent study showed that NOD1 expression was weakly positive in the cytoplasm of odontoblasts in specimens without carious lesion but was upregulated in the odontoblastic layer of specimens with carious lesion [11]. However, little is known about the functional roles of NODs and the cell signaling pathways through NODs in odontoblasts. It has been considered that human primary odontoblasts have difficulty in passage culture because of replicative senescence and too little cells isolated from dental pulp. As an alternative human primary odontoblast, we used KN-3 cells, which are an established rat odontoblastic cell line, and express odontoblastic cell markers as well as high levels of alkaline phosphatase activity. In this study, we demonstrated that the intracellular expression level of NOD1 was stronger than that of NOD2 (Figure 1(a)) and NOD1-specific ligand and TNF-*α* significantly induced the production of chemokines, such as CINC-1, CINC-2, MCP-1, and CCL20 (Figure 2). These findings suggest that NOD1 dominantly expressed in KN-3 cells as PRR functions to upregulate chemokines as proinflammatory mediators. As dental caries progresses to the odontoblast layer, there is a transition from gram-positive aerobic bacteria in early caries to anaerobic gram-negative bacteria in deep carious lesions [25–27]. Regarding the progression of dental caries, our findings suggest that NOD1 expressed in odontoblasts detects peptidoglycan of gram-negative bacteria, such as iE-DAP, and induces the production of several chemokines as proinflammatory mediators of dental pulp inflammation.

The CINC family is the rat counterpart of the human GRO protein, and CINC-1 has high homology with human IL-8 [28, 29] and neutrophil chemotactic activity [30]. MCP-1 and CCL20 recruit monocytes and lymphocytes into inflammatory lesions, respectively [31, 32]. Recent studies have reported that significantly higher levels of IL-8 and TNF-*α* were detected in caries-exposed pulps and irreversible pulpitis as compared with normal teeth [33, 34], and the production of MCP-1 as an inflammatory mediator was also significantly increased in reversible and irreversible stages of pulp inflammation compared with the control [33]. Moreover, we previously reported that the level of CCL20 mRNA expression in inflamed pulp tissues was higher than that in clinically normal pulp tissues and CCL20-expressing cells in the dental pulp were identified [35, 36]. Regarding inflammatory aspects of dental pulp beneath deep dental caries, previous studies reported that colocalization of macrophages with mature dendritic cells and CD4^+^ T cells was observed in deep dental caries [37], and CD68, which is strongly expressed by the macrophages, was positive in the inflamed pulp tissue, but the number of the macrophages present in the normal pulp tissue was very small [38]. Our* in vitro* experiment demonstrated that CCL20 expression was induced in both monocytes and macrophages after caries-related bacterial exposure, and HDPFs were also produced CCL20 in response to proinflammatory cytokines [35, 36]. These previous findings suggest that chemokines, such as IL-8, MCP-1, and CCL20, might play an important role in the progression of pulpitis via recruitment of inflammatory cells into the dental pulp. Therefore, our results suggest that NOD1 expressed in odontoblasts plays an important role in the progression of pulpitis because of the ability for chemokines upregulation.

This study also revealed that the intracellular expression level of NOD2 was lower than that of NOD1 by flow cytometric analysis but NOD2 expressed in KN-3 cells could not function properly, because MDP, NOD2-specific ligand, could not induce the expression and production of CINC-1, CINC-2, MCP-1, and CCL20 (Figures 2(a) and 2(b)). However, there is the possibility that NOD2 in KN-3 cells upregulates inflammatory mediators other than the chemokines plotted on the cytokine antibody array. A previous report demonstrated that odontoblasts and pulp fibroblasts differ in their innate immune responses to PAMPs such as LTA, LPS, and polyinosinic-polycytidylic acid (Poly(I:C)) [39]. We previously demonstrated that NOD2 and TLR2 are functionally predominant receptors stimulating the production of proinflammatory mediators, such as IL-8, IL-6, MCP-1, and PGE_2_ in HDPFs [8]. These findings suggested that odontoblasts and dental pulp fibroblasts may play different roles in bacterial recognition in innate immunity.

In this study, we also investigated iE-DAP- or TNF-*α*-induced signaling cascades leading to increased production of chemokines in KN-3 cells. Our results showed that the iE-DAP-activated p38-AP-1 pathway increased the production of CINC-2 and CCL20, and ERK-NF-*κ*B pathway activation by TNF-*α* stimulation upregulated the production of CINC-2 (Figures 3 and 4). It has been reported that the NOD1 signal transduction pathway led to AP-1 activation via MAPK phosphorylation [20, 40] as well as NF-*κ*B activation via RICK [41, 42]. Therefore, our findings infer that the NOD1-p38-AP-1 signal pathway in KN-3 cells is characteristic of odontoblasts involved in chemokine upregulation. Our findings appear to be in agreement with the previous study, which showed that NOD1 expression was weakly positive in the cytoplasm of odontoblasts in specimens without carious lesion but was upregulated in the odontoblastic layer of specimens with carious lesion and the activation of p38 MAPK is involved in NOD-1-induced production of chemokines such as IL-8 and MCP-1 in HDPFs [11]. However, to extrapolate our results into human pathological situation has some limitations due to the nature of rodent cell line, not human. Therefore, our findings encourage us to further determine the expression profiles and functions of PRRs in human odontoblasts and their cell signaling pathways in dental pulp innate immunity and to elucidate the cause and pathologic condition of human dental pulpitis.

## 5. Conclusions

Our results strongly suggest that NOD1 expressed in odontoblasts transmits signals to the nucleus via the p38-AP-1 pathway and, therefore, may play important roles in the initiation and progression of pulpitis. Furthermore, these findings may also provide understanding of the mechanisms underlying PAMP-induced innate immune responses in odontoblasts and lead to the development of new therapeutic strategies and treatments for pulpitis.

## Figures and Tables

**Table 1:** Oligonucleotide sequences of primers used for quantitative real-time PCR.

Target gene	Accession number	Primer	Sequence
CINC-1	M86536.1	Sense	5′-cacactccaacagagcacca-3′
		Antisense	5′-tgacagcgcagctcattg-3′

CINC-2*α*	D87927.1	Sense	5′-ggctcctcaatgctgcac-3′
		Antisense	5′-ggccacaacagtccctga-3′

MCP-1	M57441.1	Sense	5′-cgtgctgtctcagccagat-3′
		Antisense	5′-ggatcatcttgccagtgaatg-3′

CCL20	U90447.1	Sense	5′-ggggtactgctggcttacct-3′
		Antisense	5′-ggcagcagtcaaagttgctt-3′

GAPDH	AF106860.2	Sense	5′-actcccattcttccacctttg-3′
		Antisense	5′-tgtagccatattcattgtcatacc-3′

CINC-1, cytokine-induced neutrophil chemoattractant-1; CINC-2*α*, cytokine-induced neutrophil chemoattractant-2*α*; MCP-1, monocyte-chemoattractant protein-1; CCL20, C-C motif chemokine ligand 20; GAPDH, glyceraldehyde 3-phosphate dehydrogenase.

## References

[1] Takeda K., Akira S. (2005). Toll-like receptors in innate immunity. *International Immunology*.

[2] Chamaillard M., Hashimoto M., Horie Y. (2003). An essential role for NOD1 in host recognition of bacterial peptidoglycan containing diaminopimelic acid. *Nature Immunology*.

[3] Inohara N., Ogura Y., Fontalba A. (2003). Host recognition of bacterial muramyl dipeptide mediated through NOD2. Implications for Crohn's disease. *The Journal of Biological Chemistry*.

[4] Huang G. T.-J., Potente A. P., Kim J.-W., Chugal N., Zhang X. (1999). Increased interleukin-8 expression in inflamed human dental pulps. *Oral Surgery, Oral Medicine, Oral Pathology, Oral Radiology, and Endodontics*.

[5] Durand S. H., Flacher V., Roméas A. (2006). Lipoteichoic acid increases TLR and functional chemokine expression while reducing dentin formation in in vitro differentiated human odontoblasts. *The Journal of Immunology*.

[6] Horst O. V., Horst J. A., Samudrala R., Dale B. A. (2011). Caries induced cytokine network in the odontoblast layer of human teeth. *BMC Immunology*.

[7] Levin L. G., Rudd A., Bletsa A., Reisner H. (1999). Expression of IL-8 by cells of the odontoblast layer in vitro. *European Journal of Oral Sciences*.

[8] Hirao K., Yumoto H., Takahashi K., Mukai K., Nakanishi T., Matsuo T. (2009). Roles of TLR2, TLR4, NOD2, and NOD1 in pulp fibroblasts. *Journal of Dental Research*.

[9] Hirao K., Yumoto H., Nakanishi T. (2010). Tea catechins reduce inflammatory reactions via mitogen-activated protein kinase pathways in toll-like receptor 2 ligand-stimulated dental pulp cells. *Life Sciences*.

[10] Veerayutthwilai O., Byers M. R., Pham T.-T. T., Darveau R. P., Dale B. A. (2007). Differential regulation of immune responses by odontoblasts. *Oral Microbiology and Immunology*.

[11] Lee Y.-Y., Chan C.-H., Hung S.-L., Chen Y.-C., Lee Y.-H., Yang S.-F. (2011). Up-regulation of nucleotide-binding oligomerization domain 1 in inflamed human dental pulp. *Journal of Endodontics*.

[12] Lin Z.-M., Song Z., Qin W. (2009). Expression of nucleotide-binding oligomerization domain 2 in normal human dental pulp cells and dental pulp tissues. *Journal of Endodontics*.

[13] Nomiyama K., Kitamura C., Tsujisawa T. (2007). Effects of lipopolysaccharide on newly established rat dental pulp-derived cell line with odontoblastic properties. *Journal of Endodontics*.

[14] Qin C., Brunn J. C., Cadena E., Ridall A., Butler W. T. (2003). Dentin sialoprotein in bone and dentin sialophosphoprotein gene expressed by osteoblasts. *Connective Tissue Research*.

[15] Nakashima M. (1992). The effects of growth factors on DNA synthesis, proteoglycan synthesis and alkaline phosphatase activity in bovine dental pulp cells. *Archives of Oral Biology*.

[16] Chen S., Rani S., Wu Y. (2005). Differential regulation of dentin sialophosphoprotein expression by Runx2 during odontoblast cytodifferentiation. *The Journal of Biological Chemistry*.

[17] Washio A., Kitamura C., Morotomi T., Terashita M., Nishihara T. (2012). Possible involvement of Smad signaling pathways in induction of odontoblastic properties in KN-3 cells by bone morphogenetic protein-2: a growth factor to induce dentin regeneration. *International Journal of Dentistry*.

[18] Hiyama T., Ozeki N., Mogi M. (2013). Matrix metalloproteinase-3 in odontoblastic cells derived from Ips cells: unique proliferation response as odontoblastic cells derived from ES cells. *PLoS ONE*.

[19] Hase N., Ozeki N., Hiyama T. (2015). Products of dentin matrix protein-1 degradation by interleukin-1*β*-induced matrix metalloproteinase-3 promote proliferation of odontoblastic cells. *BioScience Trends*.

[20] Allison C. C., Kufer T. A., Kremmer E., Kaparakis M., Ferrero R. L. (2009). *Helicobacter pylori* induces MAPK phosphorylation and AP-1 activation via a NOD1-dependent mechanism. *The Journal of Immunology*.

[21] Pääkkönen V., Rusanen P., Hagström J., Tjäderhane L. (2014). Mature human odontoblasts express virus-recognizing toll-like receptors. *International Endodontic Journal*.

[22] Horst O. V., Tompkins K. A., Coats S. R., Braham P. H., Darveau R. P., Dale B. A. (2009). TGF-*β*1 inhibits TLR-mediated odontoblast responses to oral bacteria. *Journal of Dental Research*.

[23] Farges J.-C., Carrouel F., Keller J.-F. (2011). Cytokine production by human odontoblast-like cells upon Toll-like receptor-2 engagement. *Immunobiology*.

[24] Keller J.-F., Carrouel F., Staquet M.-J. (2010). Expression of NOD2 is increased in inflamed human dental pulps and lipoteichoic acid-stimulated odontoblast-like cells. *Innate Immunity*.

[25] Martin F. E., Nadkarni M. A., Jacques N. A., Hunter N. (2002). Quantitative microbiological study of human carious dentine by culture and real-time PCR: association of anaerobes with histopathological changes in chronic pulpitis. *Journal of Clinical Microbiology*.

[26] Love R. M., Jenkinson H. F. (2002). Invasion of dentinal tubules by oral bacteria. *Critical Reviews in Oral Biology and Medicine*.

[27] Hoshino E. (1985). Predominant obligate anaerobes in human carious dentin. *Journal of Dental Research*.

[28] Watanabe K., Konishi K., Fujioka M., Kinoshita S., Nakagawa H. (1989). The neutrophil chemoattractant produced by the rat kidney epithelioid cell line NRK-52E is a protein related to the KC/gro protein. *The Journal of Biological Chemistry*.

[29] Nakagawa H., Komorita N., Shibata F. (1994). Identification of cytokine-induced neutrophil chemoattractants (CINC), rat GRO/CINC-2*α* and CINC-2*β*, produced by granulation tissue in culture: purification, complete amino acid sequences and characterization. *Biochemical Journal*.

[30] Shibata F., Konishi K., Kato H. (1995). Recombinant production and biological properties of rat cytokine-induced neutrophil chemoattractants, GRO/CINC-2*α*, CINC-2*β* and CINC-3. *European Journal of Biochemistry*.

[31] Hieshima K., Imai T., Opdenakker G. (1997). Molecular cloning of a novel human CC chemokine liver and activation- regulated chemokine (LARC) expressed in liver. Chemotactic activity for lymphocytes and gene localization on chromosome 2. *The Journal of Biological Chemistry*.

[32] Carr M. W., Roth S. J., Luther E., Rose S. S., Springer T. A. (1994). Monocyte chemoattractant protein 1 acts as a T-lymphocyte chemoattractant. *Proceedings of the National Academy of Sciences of the United States of America*.

[33] Abd-Elmeguid A., Abdeldayem M., Kline L. W., Moqbel R., Vliagoftis H., Yu D. C. (2013). Osteocalcin expression in pulp inflammation. *Journal of Endodontics*.

[34] Elsalhy M., Azizieh F., Raghupathy R. (2013). Cytokines as diagnostic markers of pulpal inflammation. *International Endodontic Journal*.

[35] Nakanishi T., Takahashi K., Hosokawa Y., Adachi T., Nakae H., Matsuo T. (2005). Expression of macrophage inflammatory protein 3*α* in human inflamed dental pulp tissue. *Journal of Endodontics*.

[36] Takahashi K., Nakanishi T., Yumoto H., Adachi T., Matsuo T. (2008). CCL20 production is induced in human dental pulp upon stimulation by *Streptococcus mutans* and proinflammatory cytokines. *Oral Microbiology and Immunology*.

[37] Harmon M. A., Tew J. G., Best A. M., Hahn C.-L. (2009). Mature dendritic cells in inflamed human pulps beneath deep caries. *Oral Surgery, Oral Medicine, Oral Pathology, Oral Radiology, and Endodontology*.

[38] Manolea H., Mogoanta L., Margaritescu C., Deva V., Şurlin P., Caraivan O. (2008). Immunohistochemical aspects of the evaluation of the inflammatory answer of the dental pulp. *Romanian Journal of Morphology and Embryology*.

[39] Staquet M.-J., Durand S. H., Colomb E. (2008). Different roles of odontoblasts and fibroblasts in immunity. *Journal of Dental Research*.

[40] Strober W., Murray P. J., Kitani A., Watanabe T. (2006). Signalling pathways and molecular interactions of NOD1 and NOD2. *Nature Reviews Immunology*.

[41] Kobayashi K., Inohara N., Hernandez L. D. (2002). RICK/Rip2/CARDIAK mediates signalling for receptors of the innate and adaptive immune systems. *Nature*.

[42] Inohara N., Koseki T., Del Peso L. (1999). Nod1, an Apaf-1-like activator of caspase-9 and nuclear factor-*κ*B. *The Journal of Biological Chemistry*.

